# Single cell studies of the cell cycle and some models

**DOI:** 10.1186/1742-4682-2-4

**Published:** 2005-02-09

**Authors:** JM Mitchison

**Affiliations:** 1 Institute for Cell, Animal and Population Biology, University of Edinburgh, Edinburgh EH9 3JT, UK

## Abstract

Analysis of growth and division often involves measurements made on cell populations, which tend to average data. The value of single cell analysis needs to be appreciated, and models based on findings from single cells should be taken into greater consideration in our understanding of the way in which cell size and division are co-ordinated. Examples are given of some single cell analyses in mammalian cells, yeast and other microorganisms. There is also a short discussion on how far the results are in accord with simple models.

## Introduction

What is the point of single cell studies of the cell cycle? The simple answer is that they provide extra information that is not available from studies of cell populations. Without them a cell biologist can be misled.

It is easiest for me to start with the theme of the extensive results on single cells of the fission yeast *Schizosaccharomyces pombe *with which I have worked since the mid-1950s. It was then a fairly obscure organism for physiological studies though it had a good genetic background found by U. Leupold in Bern [1]. Since then it has flourished and quite large international meetings are now devoted entirely to it. For those unfamiliar with it, it is like a scaled-up bacterial rod with division at a medial septum, unlike budding yeasts.

One the early results on its growth came from a single cell study by Bayne-Jones and Adolph [2]. Here I need to make a small digression about references. They will be given in this article but there are much longer accounts of nearly all the topics in my recent 100-page review [3]. When I took up fission yeast in the mid-fifties, I used a new microscopic technique, which gave by optical interferometry the total dry mass of single growing cells as well as their volume [4]. Volume increased, approximately in an exponential curve, through the first three quarters of the cycle but then stayed constant for the last quarter between mitosis and division. But total dry mass increased approximately linearly through the whole cycle. This was the first demonstration of linear growth, and I was surprised.

## Early synchrony techniques by induction

This period of the fifties was when attention in this field was largely focused on the successful synchronisation of *Tetrahymena *and *Chlorella *by periodic changes in their environment. Good synchronous cultures would mean that powerful biochemical techniques, often enzyme activity assays at that time, could be applied in a cell cycle context. In the next 15 years, induction synchrony was somewhat improved but the cell cycles were always and inevitably distorted. Methods were also developed to select out a fraction of an asynchronous culture in one stage of the cycle and grow it up separately (for example," membrane elution", where cells growing on a membrane come away at division). They produce less distortion but a much lower yield than induction.

Because of what can be measured in synchronous cultures, they are the natural choice for the molecular biologist. But it is as well to remember their limitations. The distortions after induction have been mentioned, but even with selection synchrony there are problems. The main one is that they are, in practice, not all that synchronous. The selected cells come from more than a very narrow region of the cycle. Some of the variation can be reduced by a correction for asynchrony [5] but there is still cell-to-cell variation in cycle stage and this can obscure the fine detail of the cycle. Single cell measurements may help here.

## Single cell analysis in yeast

Returning to single cell analyses of fission yeast, volume growth was followed in finer detail by Mitchison and Nurse [6]. One part of this analysis, on films taken previously by Fantes [7], showed that increase in volume was not a simple exponential during the growth phase in the first three quarters of the cycle but rather two linear segments with a rate change point (RCP) between them. The position of the RCP showed a large cell-to-cell variation. An important moral here is that these two linear segments vanished into an apparent exponential increase in a "well synchronised" culture made by selection. Such a culture scarcely showed the plateau in growth during the last quarter of the cycle. This distinction between single cells and synchronous cultures does of course depend on the frequency and accuracy of the data points. If the points have too much scatter, the fine detail of the single cell linear patterns is lost. There is also a second RCP at the end of the cycle.

A much more detailed analysis of populations of single cells followed on films was made by Sveiczer *et al. *[8] on fission yeast. A plot of extension growth against birth size has a strong negative slope. So also does a plot of cycle time against birth size. This has important implications for the definitions of "size control", discussed in that paper.

## Problems of single cell analysis

Single cell studies have their problems. We have been lucky in using yeasts that are not apparently affected by growing on warm agar pads under a coverslip. They show "balanced growth", a property in which there is no change in extensive properties between successive cycles [9] and that should always be checked. Useful deductions can often be made with unbalanced growth but it will be a distortion of the normal cycle.

The cells also have to keep still or be followed, a problem discussed below. We have not found ways of sticking yeast to glass (e.g. with lectins) that permit "normal" growth. Cells may also need a continuous supply of fresh medium, probably for oxygenation. Various types of microscopic mounting chambers have been described in the last 50 years or so, e.g. [10], but few seem to have been stringently tested.

Many experimental studies on cell growth kinetics can be tedious; single cell studies are no exception. Here, however, modern automation is beginning to have very promising prospects. Anyone who has spent a day on a yeast film re-focusing the microscope every 5 min will welcome auto-focusing devices that are now available. Analysis has also become much easier with electronic imaging followed by image analysis programmes, and perhaps presentation on spreadsheets. It is now possible to have a programme that requires some hand work in the initial setting up under the microscope but will then run automatically, measuring cell length and diameter. This has been done for fifty or more single cells of fission yeast – a long way from the early days of using a ruler to measure the length of yeast cells on projected film images. Another point that should be raised here is that the new technology could profitably be applied to the growth of *Escherichia coli*. The limitations of synchronous cultures in hiding the fine detail of increases in volume or area could well mean that single cell studies might reveal more than an exponential increase. There might even be something like the two linear patterns that were popular models in earlier work with this bacterium [11].

## What to measure

Volume and area of a rod-shaped organism are two of the parameters that can be measured in single growing cells. So is dry mass by interferometry. But there others, of which one of the most interesting is the use of the Cartesian diver, which was originally developed some fifty years ago at the Carlsberg Laboratory in Copenhagen. It requires technical skills and very tightly controlled temperature in water baths, but it is exquisitely sensitive. It can be used in at least two ways. One is as a diver balance, which measures "reduced weight" or weight in water. Providing there are not major changes in chemical composition, this is proportional to total dry mass. It was used on single cells of *Amoeba proteus *in an important classic paper by Prescott [12] mentioned below. It can also be used with minute divers as a respirometer. Hamburger [13] measured oxygen uptake in *Acanthamoeba a*nd CO_2 _production in fission yeast (Hamburger *et al. *[14]), in both cases over several cell cycles starting with single cells – a remarkable achievement. In both cases, the results were elegant linear patterns with an RCP at division.

Another interesting single cell method was the colorimetric enzyme assay of single yeast cells in microdrops [15]. This might have been developed with promise, but was not followed up, partly perhaps because the results differed from similar assays in synchronous cultures.

One of the advantages of single cell work with yeasts is that they stay still on an agar pad so they can be followed for a couple of cycles before overlapping spoils the image. This is not true of many mammalian cells, which move around on the substrate. One solution to this problem comes in the work on fibroblasts (mouse L cells) described in Zetterberg [16], Killander and Zetterberg [17], Zetterberg and Killander, [16]. These are part of an impressive body of work initiated using optical machinery gathered by Trigvar Caspersson, along with a great deal of skill and hard work. In one set of experiments on single cells [17], they made a measurement of the dry mass of single cells by interferometry and then placed it in the cycle by following it as it moved about until it divided. The difference in timing between the measurement and cell division gave the timing in the cycle. A second set of experiments used frequency analysis to set the cycle stages. This is a method widely used to determine G1, S and G2 in flow cytometry but is less suitable for the slow and imprecise doublings in something like dry mass. I therefore regard the single cell analyses as more reliable and they are not the same as those from the second method.

What are needed now are techniques that combine the subtlety and precision of single cell measurements with the new techniques of automation. A promising start was made by Zicha and Dunn [19], and the development is being actively pursued elsewhere.

Organisms which tend to be forgotten about these days are those lower eukaryotes that make poor material for molecular biologists because of inadequate genetic backgrounds. One important set of results are those from the early pioneer work of Prescott [12] on *Amoeba proteus *mentioned above. The results showed that the increase of single cell "dry mass" fell in a reverse exponential, with a rapid increase at the start of the cycle falling to zero towards the end. This, of course, is lethal for anyone who believes that a rising exponential is the paradigm for the cell cycle.

*Tetrahymena pyriformis *has a long and distinguished history in the cell cycle with its early induction synchrony. But in the 1960s there was a burst of studies on selected single cells or small groups. The growth patterns were often not well defined but it seems that absolute measurements of volume and of respiration rate were a better fit to linear growth (Prescott, [20]). Such analyses might now be checked using some of the semi-automated procedures referred to above.

## Growth in syncytia

*Physarum polycephalum *is a myxomycete of considerable importance in some earlier work on cell cycle control. It is effectively a big single multinucleate cell with complete natural synchrony in nuclear division. It does not show exponential increase in macromolecular synthesis. For instance, there are two peaks in the rate of protein synthesis, one in the S period and the other in G2 (Mittermayer *et al*, [21]).

## General conclusion

It would appear that there are no universal patterns of growth in these lower eukaryotes.

## Models

My title makes mention of "some models". Let me be clear that there are two quite different types of cell cycle models. One type includes detailed mathematical and molecular models dealing with discrete periodic events like mitosis (e.g. [22]). These are complex and can illustrate the relations between many components of a network at the event, on reasonable assumptions. They are important aids in understanding the events and are a fairly recent development in the cell cycle world. There are certain limitations at present. With mitosis, the models have problems with the starting event (a size control?), with location in cellular compartments, and with the final mechanical events. However, such models will certainly develop.

However, what I am concerned with here are much earlier and much simpler models, not of periodic events in the cycle like DNA synthesis, but of continuous growth. Here the two dominant models were, for simplicity, an exponential pattern of increase and a linear one. My own view [3] of the earlier experiments is that, on the whole, they favour linear increase but it was also clear that some patterns, e.g. volume in fission yeast, are more complex. Linear increases with rate change points have certainly survived in fission yeast where there are no exponential increases (Table 1 in [3]) and this has revived for me an old hypothesis of "gene dosage". What, for instance, happens to synthesis rates between G1 and G2? But one thing is clear – that a single unifying dream of exponential synthesis is not in accord with the facts. It is really useless to wave Occam's Razor around. The end of his razor blade is "without necessity". In all reasonable judgements, the necessity is there. Beyond that is prejudice.

**Figure 1:**
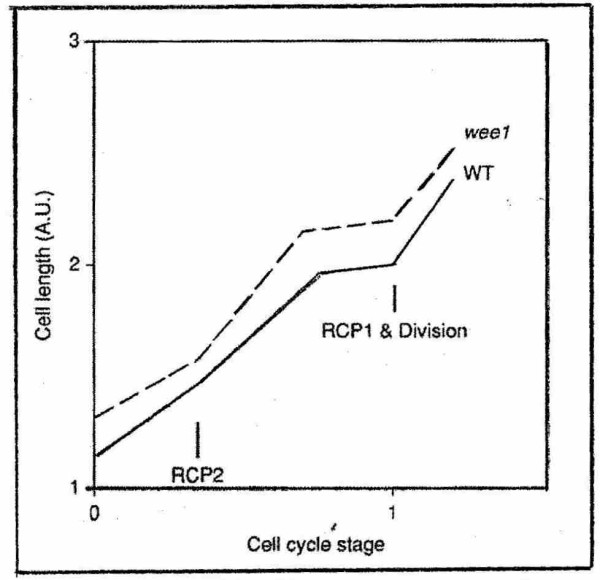
Modes of growth in cell length of wild-type and wee1 mutant cells of fhe fission yeast *Schizosaccharomyces pombe*, after Sweiczer et al. (1996)

**Figure 2:**
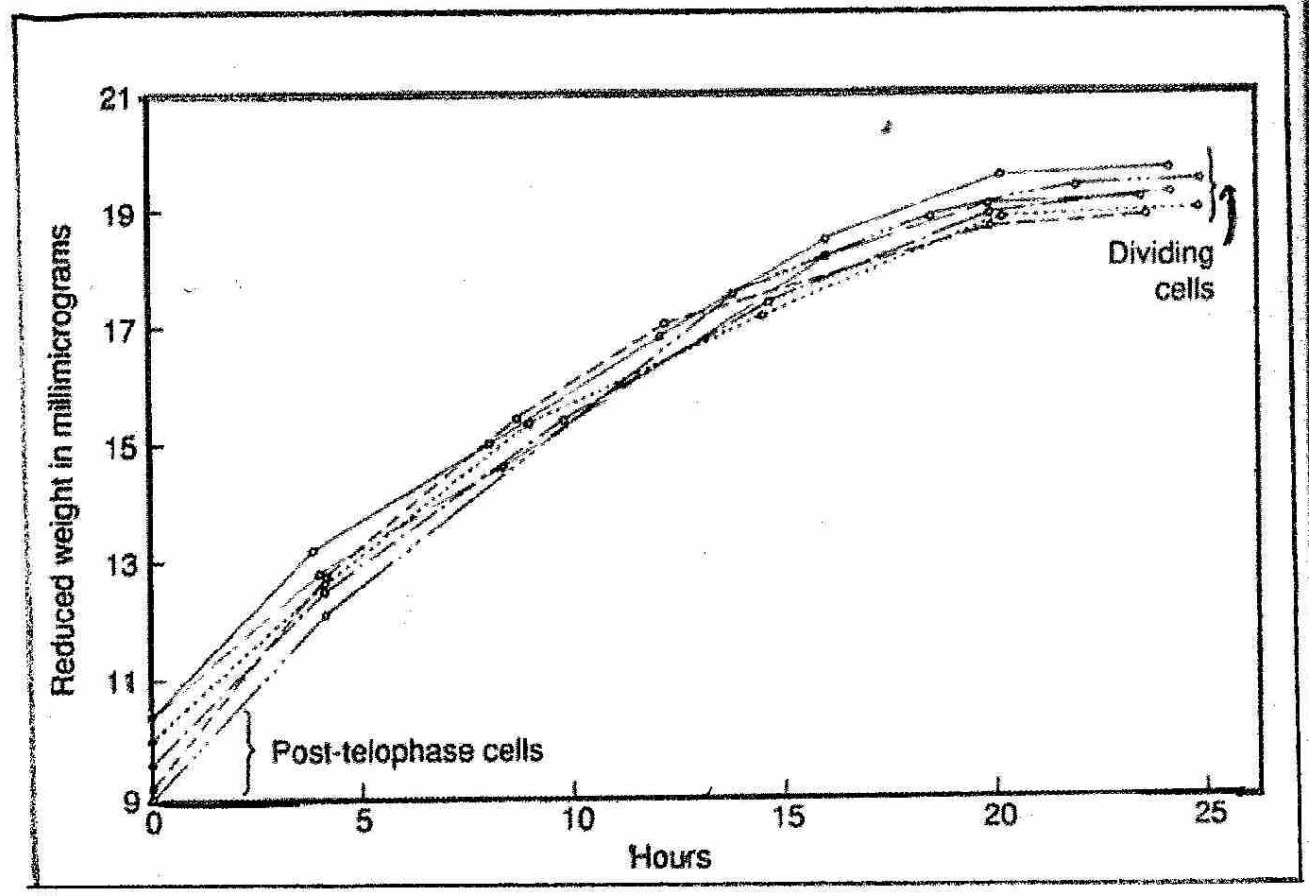
Growth through one cycle of individual Amoeba, after Prescott (1976)
